# Nine-factor-based immunohistochemistry classifier predicts recurrence for early-stage hepatocellular carcinoma after curative resection

**DOI:** 10.1038/s41416-020-0864-0

**Published:** 2020-05-07

**Authors:** Wei-Ren Liu, Meng-Xin Tian, Zheng Tang, Yuan Fang, Yu-Fu Zhou, Shu-Shu Song, Xi-Fei Jiang, Han Wang, Chen-Yang Tao, Pei-Yun Zhou, Wei-Feng Qu, Zhen-Bin Ding, Yuan-Fei Peng, Jian Zhou, Jia Fan, Ying-Hong Shi

**Affiliations:** 1 Department of Liver Surgery and Transplantation, Liver Cancer Institute, Zhongshan Hospital, Fudan University; Key Laboratory of Carcinogenesis and Cancer Invasion of Ministry of Education, Shanghai, China; 2 Institutes of Biomedical Sciences, Fudan University, Shanghai, China; 3 Shanghai Key Laboratory of Organ Transplantation, Shanghai, China; 4 State Key Laboratory of Genetic Engineering and Collaborative Innovation Center for Genetics and Development, School of Life Sciences, Fudan University, Shanghai, China

**Keywords:** Hepatocellular carcinoma, Prognostic markers

## Abstract

**Background:**

Immunoscore have shown a promising prognostic value in many cancers. We aimed to establish and validate an immune classifier to predict survival after curative resection of hepatocellular carcinoma (HCC) patients who have undergone curative resection.

**Methods:**

The immunohistochemistry (IHC) classifier assay was performed on 664 patients with Barcelona Clinic Liver Cancer (BCLC) stage 0 or A HCC. A nine-feature-based HCC-IHC classifier was then constructed by the least absolute shrinkage and selection operator method. The associations between the HCC-IHC classifier and patient outcomes were assessed. Herein, a nomogram was generated from the Cox regression coefficients and evaluated by decision curve analysis.

**Results:**

We constructed an HCC-IHC classifier based on nine features; significant differences were found between the low-HCC-IHC classifier patients and high-HCC-IHC classifier patients in the training cohort in the 5-year relapse-free survival rates (46.7% vs. 26.7%, respectively; *P* < 0.001). The HCC-IHC classifier-based nomogram presented better accuracy than traditional staging systems.

**Conclusions:**

In conclusion, the HCC-IHC classifier could effectively predict recurrence in early-stage HCC patients and supplemented the prognostic value of the BCLC staging system. The HCC-IHC classifier may facilitate patient decision-making and individualise the management of postoperative patients with early-stage HCC.

## Background

Although imperative developments have been made in the early diagnosis of hepatocellular carcinoma (HCC), there has not been a relative advance in the prediction of patient recurrence.^1^ In accordance with other solid tumours, the staging of HCC largely relies on the histopathological criteria of tumour number, tumour diameter, vascular invasion, liver function, and performance status score (modified Barcelona Clinic Liver Cancer (BCLC) staging system).^2,3^ This anatomy-based system offers practical but imprecise prognostic information. Given their moderate prediction accuracy, new strategies to stratify cancer patients that have focussed on tumour cell-, tumour mutational burden-, signalling pathway-, and gene expression-based classifications are of significant usefulness,^4–7^ new strategies focussing on tumour microenvironment are also urgently needed for the advancement of clinical outcome prediction to supplement the current staging system.^8^

In situ immune cell infiltration in tumours is relevant for accurate prognostic predictions.^9–11^ In HCC, we have shown that innate and adaptive immune components, such as regulatory T cells (Tregs), macrophages, and neutrophils, in the tumour and invasive margin were strongly correlated with overall survival (OS) and relapse-free survival (RFS).^12^ We have also proposed that the intratumoural immune contexture (density, composition, location, and functional state of immune cells) could be a new predictor of prognosis and provide a novel target for an optimal immunotherapy.^13^ As a result, the immune contexture was explored to tailor individual treatments and monitor the responses to anticancer therapies.^9^

Currently, new data have revealed that Immunoscore, which is a classification based on the extent of tumour invasion by immune cells, provides a more accurate prognosis.^14^ In an international consortium led by the Society for Immunotherapy of Cancer, the densities of CD3^+^ and CD8^+^ T cells within the tumour and its invasive margin were summarised as Immunoscore, which showed the highest contribution of all clinical parameters to the recurrence risk and provided a relative estimate of the risk of recurrence in patients with colon cancer; thus Immunoscore was implemented as a new component of the tumour–nodes–metastasis (TNM)-Immune staging system.^15,16^ The Immunoscore classifier could effectively predict recurrence and survival in gastric cancer and was an ideal complementation to the TNM staging system.^17^ However, the characteristics of the HCC–immunohistochemistry (IHC) classifier are largely unknown.

Therefore, in this study, we used the least absolute shrinkage and selection operator (LASSO) Cox regression model to construct a novel HCC-IHC classifier to predict OS and RFS after surgery. The predictive performance of the HCC-IHC classifier was determined using time-dependent receiver operating characteristic (ROC) curves. Further, a nomogram that integrated the HCC-IHC classifier and four clinicopathological risk factors was established.

## Materials and methods

### Patients and database

The records of the patients with HCC who underwent primary tumour resection at Zhongshan Hospital, Fudan University (Shanghai, China) between 2005 and 2008 were reviewed. Their histopathological and clinical characteristics were scored according to the BCLC system (Table 1). Two independent cohorts, one with 195 patients (training cohort) and the other with 114 patients (testing cohort), were randomly selected from the patients treated between 2005 and 2006. A validation cohort of 355 patients was randomly chosen from those treated between 2007 and 2008. The ethical implications were approved by an ethical review board of Zhongshan Hospital.

**Table 1:** Demographic, clinical, and tumour characteristics of patients with hepatocellular carcinoma.

Patient demographics	Training cohort (*n* = 195)	Testing cohort (*n* = 114)	Validation cohort (*n* = 355)	*P* value
Age, years				
<60	150	88	263	0.68
≥60	45	26	92	
Sex (male), *n* (%)	158 (81.0%)	96 (84.2%)	306 (86.2%)	0.28
HBV	163	95	298	0.51
Liver cirrhosis, yes (%)	155 (79.5%)	89 (78.1%)	302 (85.1%)	0.12
AFP, ng/ml	76.0 (6.0, 807.0)	120.5 (5.3, 675.0)	77.0 (5.0, 861.5)	0.86
Albumin, g/dl	4.2 (3.9, 4.6)	4.4 (4.1, 4.6)	4.4 (4.0, 4.7)	0.02
Bilirubin, μmol/l	15.1 (11.9, 19.0)	15.0 (11.7, 19.4)	14.1 (10.5, 18.4)	0.05
ALT, IU/l	41.0 (27.5, 67.5)	41.0 (27.5, 57.5)	38.0 (26.0, 52.0)	0.10
GGT, U/l	53.0 (34.0, 100.0)	50.0 (30.3, 95.5)	57.0 (37.0, 99.0)	0.22
Tumour diameter, cm	4.0 (2.5, 6.0)	4.0 (2.5, 7.0)	3.5 (2.5, 6.0)	0.41
Microvascular invasion (yes), *n* (%)	62 (31.8%)	32 (28.1%)	98 (27.6%)	0.57
Tumour differentiation				
I–II	147	87	253	0.42
III–IV	48	27	102	
CGLC stage				0.30
Ia	128	76	254	
Ib	67	38	101	
BCLC				
0	29	13	46	0.67
A	166	101	309	

Values are presented as no. (%) or median (Q1, Q3).

*HBV* hepatitis B virus, *AFP* α-fetoprotein, *ALT* alanine aminotransferase, *GGT* γ-glutamyl transpeptidase, *CGLC* China Guideline for Liver Cancer, *BCLC* Barcelona Clinic Liver Cancer.

### Tissue microarray construction

All haematoxylin and eosin-stained slides were examined by pathologists who were blinded to the clinical characteristics or outcome of the patient. Two cores were taken: one from the core of the tumour and the other from the peritumoural region as previously described. Tissue microarray construction was performed with a manual array instrument (Shanghai Biochip Co Ltd, Shanghai, China).

### Immunohistochemistry

IHC was performed using an automated staining system (BONDMAX; Leica Microsystems) with 14 immune-related antibodies. In our study, 14 prognostic immune makers were chosen according to their close relationship with recurrence and survival ((CD3, CD4, CD8, CD57, and CD68),^18–20^ (CD66b, programmed cell death protein 1 (PD-1)),^21,22^ (CD14, CXCR5)^23,24^ and (CD20, CD27, Foxp3, CD45RA and CD45RO)^25,26^) in HCC. Detailed information is provided in Supplementary Materials. An Envision^+^ system and 3, 3’-diaminobenzidine-chromogen were applied to the slides (Dako, Copenhagen, Denmark; Table S1).

To evaluate the tissue-infiltrating immune cells, three most representative and independent fields were selected and captured at ×200 magnification. Identical settings were used for each photograph. The numbers of positive staining cells were counted using a computer-automated method (Image-pro plus 6.0, Media Cybernetics Inc.) as described elsewhere.^27^ The numbers of positive staining cells were recorded, and the mean value was used for statistical analysis. For each tumour, the three representative spots showed good level of homogeneity of stained cell numbers in each tumour and peritumour region.

### Construction of the HCC-IHC classifier using the LASSO Cox regression model

The LASSO Cox analysis was adopted to select the most useful prognostic features out of all the HCC-associated immune features for predicting survival in the training cohort; R software version 3.0.1 and the “glmnet” package (R Foundation for Statistical Computing, Vienna, Austria) were used to perform the LASSO Cox model analysis.^28^

### Statistical analysis

*t* Test was used for continuous variables, and χ^2^ test was used to compare categorical variables between two groups. For the survival analysis, Kaplan–Meier method was used to analyse the correlations between variables and RFS, and log-rank test was adopted to compare survival curves. A Cox regression model was used to perform univariate and multivariate survival analyses, and nomograms were generated from Cox regression coefficients. The performance characteristics of the nomograms were explored by calibration plots. The clinical usefulness of the nomograms was evaluated by decision curve analysis (DCA). Nomograms and calibration plots were performed with the rms package of R software, and all the other statistical tests were performed with R software. The statistical significance level was set at 0.05.

## Results

### Patient characteristics, immune signatures, and HCC-IHC classifier construction

Table 1 shows the detailed clinicopathological characteristics of the training, testing, and independent validation sets. All 664 patients underwent curative surgical resection with histologically negative resection margins. The percentages of patients in the BCLC 0 and A stages were 14.9% and 85.1% in the training cohort, 11.4% and 88.6% in the testing cohort, and 13.0%, and 87.0% in the validation cohort, respectively. Aside from albumin levels, there were no significant differences among the training, testing, and validation cohorts with regard to any patient- or tumour-related covariates.

The median follow-up time was 52.2 months (interquartile range (IQR) 26.5–56.1), 53.2 months (IQR 35.3–57.1), and 42.4 months (IQR 30.8–46.3) in the training, testing, and validation cohorts, respectively. For the entire cohort, 353/664 (53.2%) patients developed tumour recurrence during the follow-up period, and 36.3% of the patients (241/664) died during follow-up. The 1-, 3-, and 5-year OS rates were 80.1%, 64.0%, and 59.2%, respectively, and the 1-, 3-, and 5-year RFS rates were 62.7%, 48.0%, and 39.6%, respectively.

**Fig. 1:**
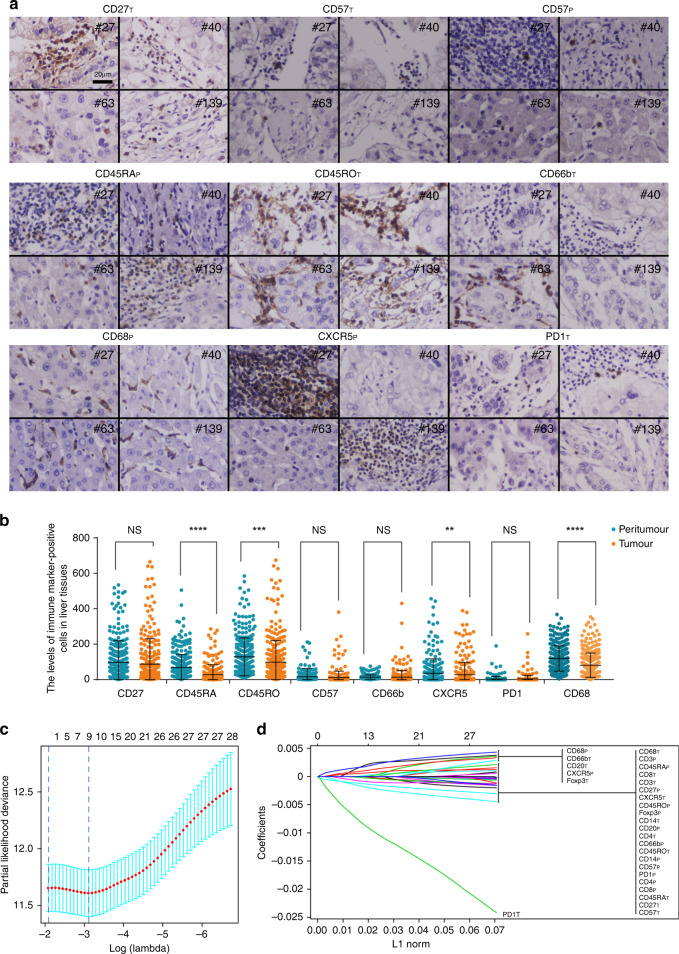
Selection of immune features by LASSO Cox analysis in hepatocellular carcinoma (HCC) patients with early stage. **a** Immunohistochemistry (IHC) staining of selected prognostic features expression in four HCC patients (#27, #40, #63, and #139), including CD27_T_, CD57_T_, CD57_P_, CD45RA_P_, CD45RO_T_, CD66b_T_, CD68_P_, CXCR5_P_, and PD-1_T_ (T represents tumoural, P represents peritumoural). Bar, 20 μm. **b** Comparison of positive immune cells in intratumoural and peritumoural tissues in IHC analysis by *t* test analysis. **c** The two dotted vertical lines were drawn at the optimal values by minimum criteria (right) and 1-s.e. criteria (left). **d** LASSO coefficient profiles of the 28 features. **P < 0.01, ***P < 0.001, and ****P < 0.0001.

IHC analysis of the samples from the 664 patients in the entire cohort showed a dominant cluster of CD8_T_ (T stands for tumoural), CD27_T_, CD4_T_, CD45RO_T_, CD27_P_ (P stands for peritumoural), CD8_P_, CD4_P_, CXCR5_T_, CXCR5_P_, CD68_P_, CD45RO_P_, and CD45RA_P_, and the expression of CD45RA, CD45RO, and CXCR5 in tumour parenchyma were less than in peritumoural tissue (Fig. 1a, b). X-tile software was used to generate the optimum cut-off densities for all 14 features in the training cohort. The LASSO Cox analysis was used to construct a prognostic classifier, which included 9 features that were mainly composed of suppressive cell markers out of the 14 features identified in the training cohort: CD57_T_, CD57_P_, CD45RO_T_, CD45RA_P_, CD27_T_, PD-1_T_, CXCR5_P_, CD68_P_, and CD66b_T_ (Fig. 1c, d). By using the LASSO Cox regression model,^17,29,30^ we then derived a formula to measure an HCC-IHC classifier for each patient based on their personal levels of the nine features: HCC-IHC classifier = (4.4601 × the level of CD45RA_P_ − 10.4116 × the level of CD27_T_ − 0.2530 × the level of CD45RO_T_ − 11.9822 × the level of CD57_T_ − 5.0658 × the level of CD57_P_ + 7.3554 × the level of CD66b_T_ + 13.9136 × the level of CD68_P_ + 7.0864 × the level of CXCR5_P_ − 61.9691 × the level of PD-1_T_) × 10^–4^. The level of each immune marker was measured as the number of stained cells positive for the specific immune marker in the tumour or peritumour tissue of the HCC tissue cores from the patient.

### Selection of prognostic predictors

In the training cohort, all variables that achieved significance at *P* < 0.05 in the univariate analysis were enrolled into the multivariate analysis with the Cox regression model. Gamma-glutamyl transpeptidase (GGT) levels (hazard ratio [HR]: 1.652; 95% confidence interval (CI), 1.117–2.441; *P* = 0.012), liver cirrhosis (HR: 1.989; 95% CI, 1.156–3.423; *P* = 0.013), and the HCC-IHC classifier (HR: 1.593; 95% CI, 1.083–2.344; *P* = 0.018) were independent prognostic factors in the multivariate Cox model (Table 2). Further, the HCC-IHC classifier was independent prognostic factors in the testing cohort (HR: 2.258; 95% CI, 1.293–3.944; *P* = 0.004) and validation cohort (HR: 2.215; 95% CI, 1.614–3.040; *P* < 0.001) (Tables S2 and S3).

**Table 2:** Univariate and multivariate cox analysis of RFS in the training cohort (*n* = 195).

Factor	RFS					
	Univariate			Multivariate		
	Hazard ratio	95% CI	*P* value	Hazard ratio	95% CI	*P* value
Sex (female vs. male)	1.467	0.874–2.462	0.147	NA		
Age, years (≤50 vs. >50)	1.165	0.792–1.713	0.437	NA		
HBsAg (negative vs. positive)	1.167	0.686–1.983	0.569	NA		
AFP, ng/ml (≤20 vs. >20)	1.248	0.844–1.845	0.267	NA		
GGT, U/l (≤54 vs. >54)	1.832	1.255–2.676	0.002	1.652	1.117–2.441	0.012
Liver cirrhosis (no vs. yes)	1.739	1.023–2.956	0.041	1.989	1.156–3.423	0.013
Tumour size, cm (≤5 vs. >5)	1.592	1.086–2.333	0.017	1.420	0.935–2.154	0.100
Microvascular invasion (no vs. yes)	1.640	1.110–2.423	0.013	1.523	0.707–3.277	0.282
Tumour differentiation (I–II vs. III–IV)	1.661	1.104–2.499	0.015	1.509	0.996–2.287	0.052
TNM stage (I vs. II)	1.713	1.133–2.590	0.011	1.107	0.492–2.490	0.805
BCLC stage (0 vs. A)	1.083	0.645–1.818	0.762	NA		
HCC-IHC classifier (high vs. low)	1.649	1.133–2.400	0.009	1.593	1.083–2.344	0.018

Cox proportional hazards regression.

*RFS* relapse-free survival, *AFP* α-fetoprotein, *ALT* alanine aminotransferase, *GGT* γ-glutamyl transpeptidase, *TNM* tumour–nodes–metastasis, *BCLC* Barcelona Clinic Liver Cancer, *HR* hazard ratio, *CI* confidential interval, *NA* not adopted.

### Performance of the HCC-IHC classifier in stratifying the recurrence risks of patients

**Fig. 2:**
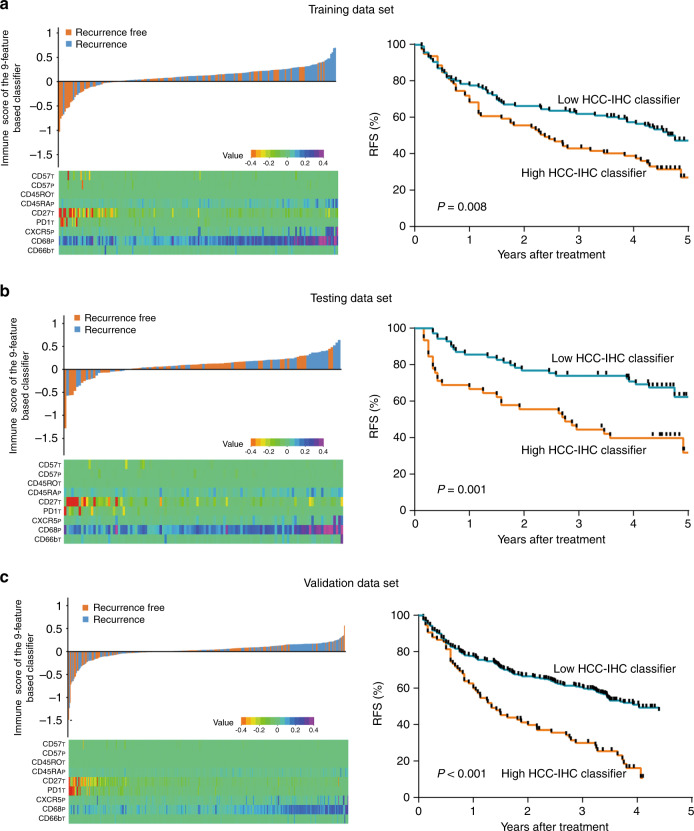
Correlation of immune cell infiltration and clinical outcome. Kaplan–Meier curves for relapse-free survival (RFS) of patients with HCC according to HCC-IHC classifier in training cohort (**a**), testing cohort (**b**), and validation cohort (**c**). *P* value was determined by log-rank test.

According to the highest *χ*^2^-value defined by Kaplan–Meier survival analysis and log-rank tests, the cut-off value was set at 0.148 with X-tile plots, ≥0.148 was considered as high, and <0.148 was considered as low in this formula.^31,32^ The distribution of the clinical characteristics did not vary significantly between the low-HCC-IHC classifier and the high-HCC-IHC classifier groups. The 5-year RFS rates were 46.7% for the low-HCC-IHC classifier group and 26.7% for the high-HCC-IHC classifier group in the training cohort (Fig. 2a). We performed the same analysis and found that the 5-year RFS rates were 62.8% for the low-HCC-IHC classifier group and 36.1% for the high-HCC-IHC classifier group in the testing cohort (Fig. 2b), with 43.9% for the low-HCC-IHC classifier group and 8.2% for the high-HCC-IHC classifier group in the internal validation cohort (Fig. 2c).

### Efficacy of the HCC-IHC classifier-based nomogram in predicting RFS

**Fig. 3:**
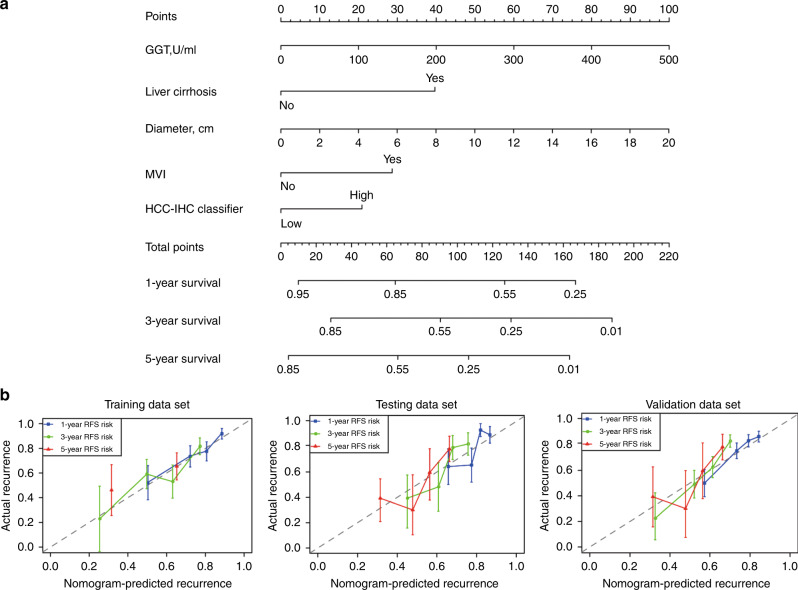
Development of the prognostic nomogram. **a** The nomogram for predicting RFS in patients after curative resection. To estimate the survival rate of an individual patient, the value of each factor is acquired on each variable axis, and a line is drawn straight upward to determine the points. The sum of these five numbers is located on the Total points axis, followed by a line drawn downward to the survival axes to determine the probability of 1-, 3-, and 5-year RFS. **b** The calibration curve for predicting RFS at 1, 3, and 5 years in training, testing, and validation cohorts. The nomogram-predicted probability of survival is plotted on the *x* axis, and the actual survival is plotted on the *y* axis.

The prognostic nomogram that integrates all significant covariates for RFS in the training cohort is shown in Fig. 3a. The *C*-index for the RFS prediction was 0.681 (95% CI, 0.624–0.739). The calibration plot for the probability of survival at 3 or 5 years after hepatectomy showed optimal agreement between the prediction made by the nomogram and the actual observation (Fig. 3b).

**Fig. 4:**
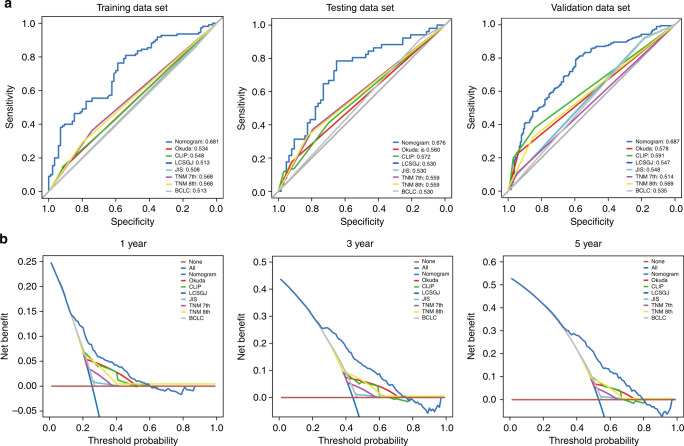
ROC curves for HCC-IHC classifier and decision curve analysis for HCC-IHC classifier-based nomogram. ROC curves for HCC-IHC classifier and seven staging systems in the training, testing and validation cohorts (**a**). Nomogram was compared to other stage systems in terms of the 1-, 3-, and 5-year RFS in the entire cohort (**b**). The horizontal solid black line represents the assumptions that no patient will experience the event, and the solid grey line represents the assumption that all patients will relapse. On decision curve analysis, nomogram showed better net benefit compared with other stage systems across a range of threshold probabilities.

Our nomogram presented better accuracy in predicting both short- and long-term survival in the training cohort than other prognostic indicators. The *C*-index of the nomogram was 0.681, which was significantly higher than those of the Okuda staging system (0.534), CLIP staging system (0.548), LCSGJ staging system (0.513), JIS staging system (0.508), Seventh TNM staging system (0.568), Eighth TNM staging system (0.568), and BCLC staging system (0.513) (Fig. 4a).

In the testing cohort, the *C*-index of the HCC-IHC classifier nomogram for the prediction of RFS was 0.676, and a calibration curve presented good agreement between the predictions and observations for the probability of 5-year survival (Table 2). The *C*-index of the HCC-IHC classifier nomogram was higher than those of the other existing staging systems for the prediction of the RFS of HCC. The *C*-indices of the other systems were as follows: Okuda (0.560), CLIP (0.572), LCSGJ (0.530), JIS (0.530), Seventh TNM (0.559), Eighth TNM staging system (0.559), and BCLC staging system (0.530) (Fig. 4a). In the validation cohort, the *C*-index of the HCC-IHC classifier nomogram for the prediction of RFS was 0.687, and a calibration curve presented good agreement between the predictions and observations for the probability of 5-year survival (Table S4). The *C*-index of the HCC-IHC classifier nomogram was higher than those of the other existing staging systems for the prediction of the RFS of HCC. The *C*-indices of the other systems were as follows: Okuda (0.578), CLIP (0.591), LCSGJ (0.547), JIS (0.548), Seventh TNM (0.514), Eighth TNM staging system (0.569), and BCLC staging system (0.535). These results again suggested that the nomogram was useful for predicting survival in HCC (Fig. 4a). In a DCA, compared with other staging systems, the nomogram showed better net benefit with a wider range of threshold probability and improved performance for predicting 1-, 3-, and 5-year RFS in the entire data set. In early-stage HCC patients, this result further represented the superior estimation of decision outcomes at higher threshold probability levels (Fig. 4b).

### Efficacy of the specificity/sensitivity of HCC-IHC classifier in predicting RFS

In order to compare for specificity/sensitivity of HCC-IHC classifier to the basic traditional predictors such as GGT in predicting RFS of HCC, we undertook specificity/sensitivity analysis of GGT, liver cirrhosis, tumour diameter, and microvascular invasion (MVI), as in the training cohort, and we found that the *C*-index of HCC-IHC classifier was 0.649, which was higher than that of liver cirrhosis (0.568), tumour diameter (0.578), and MVI (0.552) and less than GGT (0.671). In the testing cohort, the *C*-index of HCC-IHC classifier was 0.615, which was higher than that of liver cirrhosis (0.556), tumour diameter (0.598), and MVI (0.583) and equalled to GGT (0.619). In the validation cohort, the *C*-index of HCC-IHC classifier was 0.590, which was higher than that of liver cirrhosis (0.532) and MVI (0.583), equalled to GGT (0.619), but less than that of tumour diameter (0.667) and MVI (0.602) (Fig. S1). These results showed that, in contrast to traditional predictors, HCC-IHC classifier was still an ideal predictor in predicting RFS of HCC.

In order to compare for specificity/sensitivity of HCC-IHC classifier to each single marker, area under the ROC curve analyses were undertaken. As in the training cohort, we found that *C*-index of HCC-IHC classifier was higher than other markers, and the results were reduplicated in the testing and validation cohort (Fig. S2).

## Discussion

In our previous study, we found that infiltrating immune cells, such as Tregs, neutrophils, macrophages, B cells, and hepatic stellate cells, as well as the expression of immune checkpoint molecules, such as PD-L1, were associated with OS and RFS in HCC patients.^13,33^ Herein, we answered three novel questions. First, can we extend the prognostic value associated with HCC-IHC classifier to patients with early-stage HCC, such as BCLC 0 and A HCC? Second, is the HCC-IHC classifier associated with the prognosis of HCC patients? Finally, is the HCC-IHC classifier-based staging system more powerful than the existing staging system in predicting patient prognosis?

The immune contexture, which is dependent on the density, composition, functional state, and organisation of the leukocytes infiltrating the tumour, was associated with the prognosis and predicted the response to treatment.^11^ The Immunoscore, which was established based on the densities of CD3^+^ and cytotoxic CD8^+^ T cells in the tumour and the invasion margin, provided a reliable estimate of the risk of recurrence in patients with colon cancer.^15^ In gastric cancer, Immunoscore could effectively predict recurrence and survival and supplemented the prognostic value of the TNM staging system. Furthermore, Immunoscore acted as a useful tool for identifying patients who might benefit from adjuvant chemotherapy.^17^ In our study, using an IHC method, we stained for 14 markers of immune-related cells in the tumour parenchyma and peritumour area. Then nine common markers were selected, and a formula was generated. In the prognosis analysis, we found that the 5-year RFS rates were higher in the low-HCC-IHC classifier group, suggesting that an increase in the HCC-IHC classifier was correlated with an adverse prognosis. Together with GGT levels, liver cirrhosis, tumour diameter, and MVI, the HCC-IHC classifier was an independent prognostic factor in the multivariate Cox model.

LASSO is a popular method for regression of high-dimensional predictors; it has been extended and broadly applied to the Cox proportional hazard regression model for survival analysis with high-dimensional data. LASSO can also be used for optimal selection of markers in high-dimensional data with a strong prognostic value and low correlation among each other to prevent overfitting. In our study, we calculated the correlation matrix of the included features in the training data, and there was no big correlation between each pair of the selected features. Next, we calculated the variance inflation factor (VIF), a widely used statistic for the evaluation of co-linearity, of the selected features in the Cox model, and all the VIFs were <1.5, except one feature that had the VIF = 1.51 was not high to be considered that there was a co-linearity problem in our model.

Until now, the staging of HCC has been largely dependent on histopathological criteria. Similar to the BCLC staging system, the Hong Kong staging system and HCC guidelines in China include tumour number, tumour diameter, and liver function.^1,2,34^ These systems have been broadly used in clinical practice but provide incomplete prognostic information. The outcomes after therapy for patients with HCC are variable even when patients are assigned to the same BCLC stage. In colorectal cancer (CRC), many new methods to classify cancer progression have been proposed; they mostly rely on tumour cell characteristics, such as morphology, molecular pathways, mutational burden, cell origin, and gene expression patterns.^35–37^ However, other major parameters, especially the tumour microenvironment, should also be taken into consideration. Thus the TNM-Immune staging system, which is a new classification, showed its superiority in predicting the prognosis of CRC treatment.^38,39^

In HCC immunology study, OS and RFS were gradually prolonged as the Immunoscore increased.^40^ As an inflammatory modulator, TREM-1 correlated significantly with increased HCC recurrence and poorer survival.^41^ The activation status of tumour-infiltrating leukocytes was manipulated by the immunosuppressive gradient in primary HCC.^42^ Under the track of CCRL1, CCR7 (+) Treg-like cells facilitated tumour development and indicated adverse prognosis in HCC patients.^43^ In our study, the HCC-IHC classifier-based nomogram showed better accuracy in predicting both short- and long-term survival in the training, testing, and validation cohorts. We found that an increase in the HCC-IHC classifier was correlated with an adverse prognosis. As our result seems be in contrast with precious study which showed that high Immunoscore was associated with favourable prognosis, the underlying difference was that our HCC-IHC classifier was mainly composed of suppressive immune cell marker cluster, such as CD68, CD66b, and PD-1.^12,44,45^ Thus lower HCC-IHC classifier, which was in parallel with lower suppressive immune cell markers, is equal to hotter immune microenvironment or high Immunoscore, which was in line with more positive immune markers, which accentuated the finding in Galon and colleagues’ work.

Cancer immunotherapy is expanding rapidly due to the encouraging clinical results obtained with monoclonal antibodies that directly block checkpoint molecules, such as cytotoxic T-lymphocyte protein 4 (CTLA-4), PD-1, and its ligand PD-L1, that negatively regulate T cell responses.^46–48^ In HCC, the partial response rate for treatment with tremelimumab, which blocks CTLA-4, was 17.6%, and the disease control rate was 76.4%, which indicates that tremelimumab presents effective antitumour and antiviral activities.^49^ In another phase 1/2 dose escalation and expansion trial, the objective response rate was 20% in patients treated with 3 mg/kg nivolumab, which targets PD-1.^50^ Growing evidence supports Immunoscore as a prognostic biomarker for adjuvant therapy use. In gastric cancer treatment, adjuvant chemotherapy provided a better survival benefit to patients with stage II and III disease who were classified as high Immunoscore. However, the efficiency of the IHC classifier in HCC treatment guidance remains to be determined.

Our current study has limitations. First, it is a retrospective study with limited generalisability because all the patients were Chinese, and the clinical characteristics distribution might be more heterogeneous in other regions. Moreover, our study only enrolled BCLC stage 0 and A patients, making it susceptible to the inherent biases of such a study format. Second, although we assessed 14 immune markers, we could not encompass all the immune cell populations. New technological advances, such as single-cell transcriptome analysis and multiplex immunofluorescence assays, will improve the characterisation of tumour–immune interactions. Finally, our study was based on a single institution, and external validation is needed in our future work.

In conclusion, our study established a novel standardised immune-based assay for the classification of HCC. The HCC-IHC classifier can effectively predict recurrence and survival and adds prognostic value to the BCLC staging system. A nomogram that includes the HCC-IHC classifier may help predict individual recurrence risks and help facilitate clinician decision-making for early-stage HCC patients.

## Supplementary information

Supplementary Files

## Data Availability

The data sets are presented in the additional supporting files.
