# Supplementary material for: Nine-factor-based immunohistochemistry classifier predicts recurrence for early-stage hepatocellular carcinoma after curative resection
Source: Br J Cancer. 2020 May 7;123(1):92–100. doi: 10.1038/s41416-020-0864-0 (PMC7341807; doi:10.1038/s41416-020-0864-0)
Supplement: Supplementary file 1 — Supplementary Files [file 41416_2020_864_MOESM1_ESM.docx]

**DATA SUPPLEMENT**

**Nine-factor-based Immunohistochemistry Classifier that Predicts Recurrence for Early-Stage Hepatocellular Carcinoma after Curative Resection**

**SUPPLEMENTARY METHODS**

**Immunohistochemistry (IHC) and image-based analysis of immune markers.**

IHC was performed using an automated staining system (BONDMAX; Leica Microsystems) with 14 immune-related antibodies. In our study, 14 prognostic immune makers were chosen according to their close relationship with recurrence and survival (CD3 (T cell receptor antigen), CD4 (T cell receptor antigen), CD8 (co-receptor for the T cell receptor), CD57 (expressed on NK cells and T cells), and CD68 (expressed on in the monocyte lineage)), [CD66b (expressed on neutrophils and eosinophils) and programmed cell death protein 1 (PD-1) (extended CD28/CTLA-4 family of T cell regulators)] 24,25, (CD14 (expressed on most human monocytes and macrophages) and CXCR5 (a chemokine receptor expressed on B cells, central memory CD4 T cells, and T folicular helper cells)) 26,27 and (CD20 (expressed on B-cell lymphocyte), CD27 (member of the TNF-receptor superfamily), Foxp3 (expressed on regulatory T cells) and CD45RO/CD45RA (expressed on activated and memory T cells, some B cell subsets, activated monocytes/macrophages, and granulocytes)) in HCC.

For CD4, CD8, CD20, CD27 and FOXP3 analysis, epitope retrieval at 100 °C was performed in Tris-EDTA buffer, citrate buffer was selected for analysis of remaining markers. Intrinsic peroxidase activity was blocked by 3% hydrogen peroxide for 15 minutes at room temperature. A blocking buffer consisting of 5% albumin from bovine serum were used for 30 minutes. Slides were then incubated with primary antibodies against the immune markers and at specific dilutions (Table S1) overnight at 4 °C. Slides were then incubated for 60 minutes at room temperature with anti-mouse or anti-rabbit secondary antibodies (Dako, Copenhagen, Denmark). Slides were then incubated with diaminobenzidine (DAB) chromogen substrate at room temperature to detect antibody staining. Following three additional washes in deionized water, slides were counterstained with hematoxylin for 5 minutes. Negative control slides omitting the primary antibodies were included in all assays. Each experiment were duplicated for three times.

For image quantification and assessment of the intra- and peritumoral expression of the 14 immune markers, stained slides were qualified at ×200 magnification) using the ScanScope Aperio AT Turbo slide scanner (Leica Microsystems). Scanned images were visualized using the Image Pro Plus 6.0 software program (Media Cybernetics, Baltimore, MD) and analyzed using the Aperio Image Toolbox and Genie image analysis tool (Leica Microsystems).

**SUPPLEMENTARY FIGURES AND FIGURE LEGENDS**


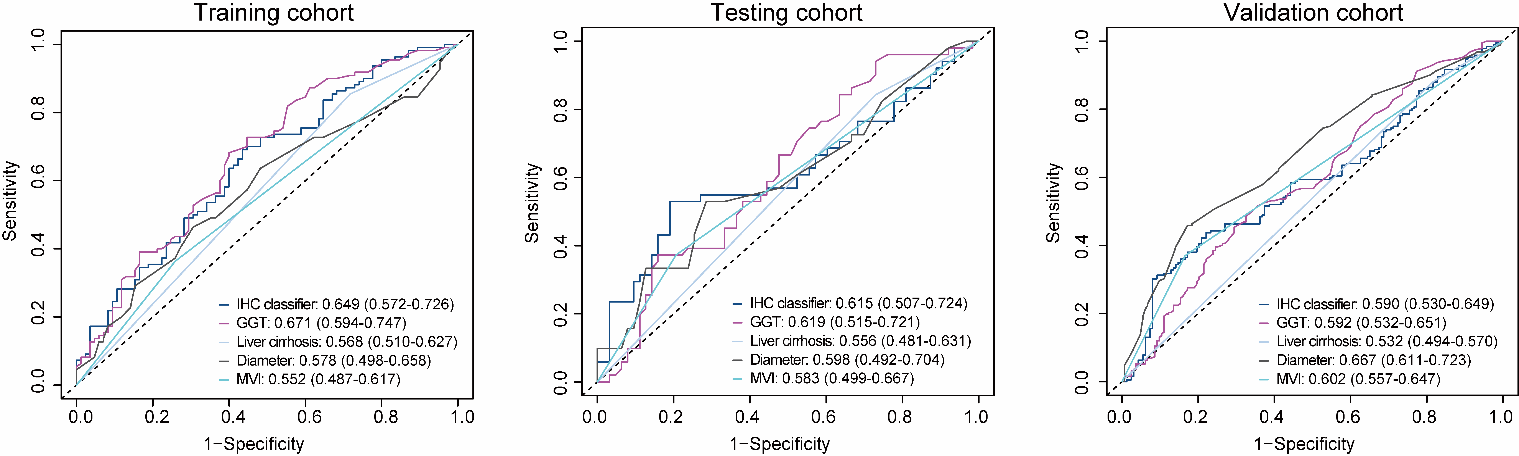


Figure S1. ROC curves for HCC-IHC classifier and other clinicopathological variate in the training, testing, and validation cohort.


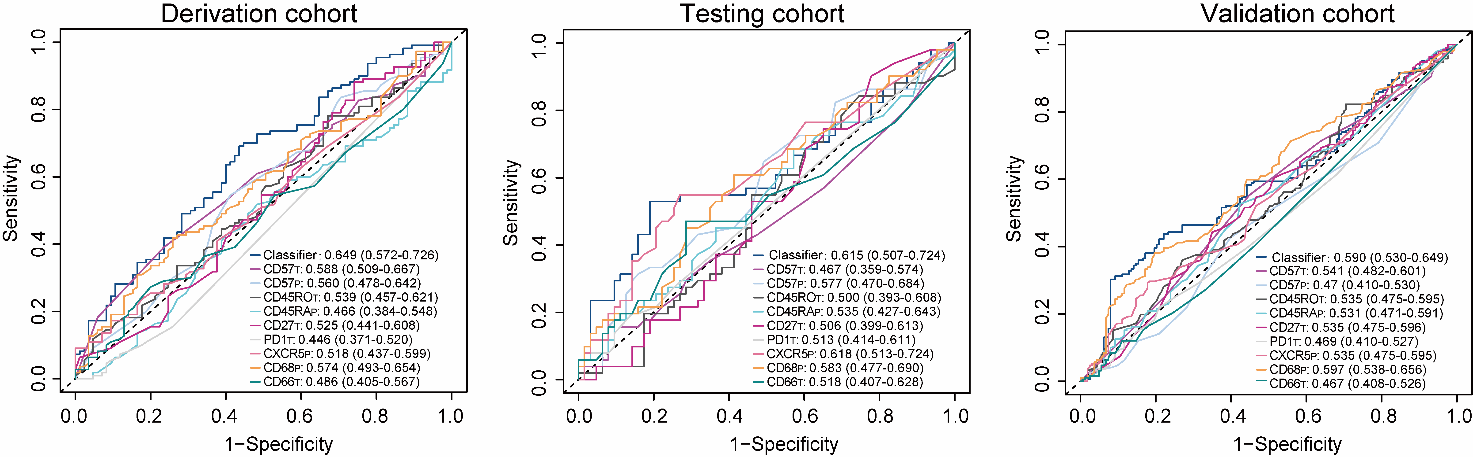


Figure S2. ROC curves for HCC-IHC classifier and each single immunological marker in the training, testing, and validation cohort.

**SUPPLEMENTARY TABLES**

Table S1. Antibody sources and staining conditions

| Markers | Antibody source | Cellular localization | Species | Clone | Antigen Retrieval | Dilution |
| --- | --- | --- | --- | --- | --- | --- |
| CD3 | DAKO | Membranous | Mouse Monoclonal | F7.2.38 | Citrate buffer (pH 6.0) | 1:200 |
| CD4 | DAKO | Membranous | Mouse Monoclonal | 4B12 | Tris/EDTA buffer (pH 9.0) | 1:400 |
| CD8 | Abcam | Membranous | Mouse Monoclonal | 144B | Tris/EDTA buffer (pH 9.0) | 1:500 |
| CD14 | Sigma | Cytoplasmic | Rabbit polyclonal | HPA001887 | Citrate buffer (pH 6.0) | 1:100 |
| CD20 | Abcam | Membranous | Rabbit polyclonal | EP459Y | Tris/EDTA buffer (pH 9.0) | 1:200 |
| CD27 | Abcam | Membranous | Rabbit Monoclonal | EPR8569 | Tris/EDTA buffer (pH 9.0) | 1:500 |
| CD45RA | Abcam | Membranous | Mouse Monoclonal | SPM504 | Citrate buffer (pH 6.0) | 1:200 |
| CD45RO | Thermo Fisher Scientific | Membranous | Mouse Monoclonal | UCHL-1 | Citrate buffer (pH 6.0) | 1:200 |
| CD57 | DAKO | Cytoplasmic | Mouse Monoclonal | TB01 | Citrate buffer (pH 6.0) | 1:200 |
| CD66b | BD | Cytoplasmic | Mouse Monoclonal | G10F5 | Citrate buffer (pH 6.0) | 1:200 |
| CD68 | DAKO | Cytoplasmic | Mouse Monoclonal | KPI | Citrate buffer (pH 6.0) | 1:200 |
| Foxp3 | Biolegend | Cytoplasmic | Mouse Monoclonal | 259D | Tris/EDTA buffer (pH 8.0) | 1:50 |
| CXCR5 | CST | Membranous | Rabbit Monoclonal | D6L3C | Citrate buffer (pH 6.0) | 1:200 |
| PD1 | CST | Cytoplasmic | Rabbit Monoclonal | D4W2J | Citrate buffer (pH 6.0) | 1:200 |

Table S2 Univariate and multivariate cox analysis of RFS in the testing cohort (n=114)

|  | RFS | | | | | |
| --- | --- | --- | --- | --- | --- | --- |
|  | Univariate | | | Multivariate | | |
| Factor | Hazard  Ratio | 95% CI | *P* value | Hazard  Ratio | 95% CI | *P* value |
| Sex (female vs. male) | 0.857 | 0.416-1.762 | 0.674 | NA |  |  |
| Age, years (≤50 vs. >50) | 1.245 | 0.706-2.197 | 0.449 | NA |  |  |
| HBsAg (negative vs. positive) | 0.903 | 0.439-1.856 | 0.782 | NA |  |  |
| AFP, ng/ml (≤20 vs. >20) | 1.446 | 0.867-2.411 | 0.158 | NA |  |  |
| GGT, U/L (≤54 vs. >54) | 1.436 | 0.826-2.495 | 0.200 | NA |  |  |
| Liver cirrhosis (no vs. yes) | 1.584 | 0.745-3.371 | 0.232 | NA |  |  |
| Tumor size, cm (≤5 vs. >5) | 2.132 | 1.220-3.726 | 0.008 | 1.924 | 1.095-3.380 | 0.023 |
| Microvascular invasion (no vs. yes) | 1.687 | 0.953-2.986 | 0.073 | NA |  |  |
| Tumor differentiation (I-II vs. III-IV) | 1.148 | 0.611-2.156 | 0.668 | NA |  |  |
| TNM stage (I vs. II) | 1.766 | 0.985-3.167 | 0.056 | NA |  |  |
| BCLC stage (0 vs. A) | 1.776 | 0.640-4.934 | 0.270 | NA |  |  |
| HCC-IHC Classifier (high vs. low) | 2.437 | 1.402-4.236 | 0.002 | 2.258 | 1.293-3.944 | 0.004 |

Abbreviations: RFS, relapse-free survival; AFP: α-fetoprotein; ALT, alanine aminotransferase; GGT, γ-glutamyl transpeptidase; TNM, tumor-nodes-metastasis; BCLC: Barcelona Clinic Liver Cancer; HR, hazard ratio; CI, confidential interval; NA, not adopted. Cox proportional hazards regression.

Table S3 Univariate and multivariate cox analysis of RFS in the validation cohort (n=355)

|  | RFS | | | | | |
| --- | --- | --- | --- | --- | --- | --- |
|  | Univariate | | | Multivariate | | |
| Factor | Hazard  Ratio | 95% CI | *P* value | Hazard  Ratio | 95% CI | *P* value |
| Sex (female vs. male) | 1.013 | 0.670-1.532 | 0.952 | NA |  |  |
| Age, years (≤50 vs. >50) | 1.185 | 0.885-1.587 | 0.254 | NA |  |  |
| HBsAg (negative vs. positive) | 1.741 | 1.158-2.617 | 0.008 | 1.706 | 1.130-2.576 | 0.011 |
| AFP, ng/ml (≤20 vs. >20) | 1.784 | 1.315-2.419 | <0.001 | 1.615 | 1.184-2.203 | 0.002 |
| GGT, U/L (≤54 vs. >54) | 1.397 | 1.049-1.860 | 0.022 | 1.211 | 0.899-1.633 | 0.208 |
| Liver cirrhosis (no vs. yes) | 1.330 | 0.860-2.058 | 0.200 | NA |  |  |
| Tumor size, cm (≤5 vs. >5) | 2.722 | 2.032-3.645 | <0.001 | 2.297 | 1.659-3.182 | <0.001 |
| Microvascular invasion (no vs. yes) | 2.062 | 1.535-2.772 | <0.001 | 1.606 | 1.053-2.450 | 0.028 |
| Tumor differentiation (I-II vs. III-IV) | 1.485 | 1.102-2.001 | 0.009 | 1.310 | 0.959-1.790 | 0.089 |
| TNM stage (I vs. II) | 1.925 | 1.420-2.608 | <0.001 | 0.978 | 0.625-1.532 | 0.923 |
| BCLC stage (0 vs. A) | 1.991 | 1.193-3.322 | 0.008 | 1.393 | 0.814-2.382 | 0.226 |
| HCC-IHC Classifier (high vs. low) | 2.328 | 1.710-3.170 | <0.001 | 2.215 | 1.614-3.040 | <0.001 |

Abbreviations: RFS, relapse-free survival; AFP: α-fetoprotein; ALT, alanine aminotransferase; GGT, γ-glutamyl transpeptidase; TNM, tumor-nodes-metastasis; BCLC: Barcelona Clinic Liver Cancer; HR, hazard ratio; CI, confidential interval; NA, not adopted. Cox proportional hazards regression.

Table S4 The comparison of nomogram with other seven staging systems in three cohort

| Staging systems | Derivation cohort | *P* value | Testing cohort | *P* value | Validation cohort | *P* value |
| --- | --- | --- | --- | --- | --- | --- |
| Nomogram | 0.681 (0.624, 0.739) |  | 0.676 (0.592, 0.759) |  | 0.686 (0.643, 0.729) |  |
| Okuda | 0.534 (0.504, 0.564) | <0.001 | 0.560 (0.519, 0.601) | 0.002 | 0.578 (0.554, 0.601) | <0.001 |
| CLIP | 0.548 (0.499, 0.596) | <0.001 | 0.572 (0.504, 0.640) | 0.02 | 0.591 (0.559, 0.624) | <0.001 |
| LCSGJ | 0.513 (0.476, 0.549) | <0.001 | 0.530 (0.481, 0.578) | <0.001 | 0.547 (0.517, 0.577) | <0.001 |
| JIS | 0.508 (0.473, 0.543) | <0.001 | 0.530 (0.481, 0.578) | <0.001 | 0.548 (0.518, 0.578) | <0.001 |
| TNM 7^th^ | 0.568 (0.524, 0.612) | <0.001 | 0.559 (0.495, 0.622) | <0.001 | 0.514 (0.494, 0.535) | <0.001 |
| TNM 8^th^ | 0.568 (0.528, 0.608) | <0.001 | 0.559 (0.499, 0.619) | <0.001 | 0.569 (0.538, 0.599) | <0.001 |
| BCLC | 0.513 (0.476, 0.549) | <0.001 | 0.530 (0.481, 0.578) | <0.001 | 0.535 (0.509, 0.562) | <0.001 |

Abbreviations: BCLC: Barcelona Clinic Liver Cancer, TNM: Tumor Node Metastasis
